# Acceptability and efficacy of vaginal self-sampling for genital infection and bacterial vaginosis: A cross-sectional study

**DOI:** 10.1371/journal.pone.0260021

**Published:** 2021-11-18

**Authors:** Claire Camus, Guillaume Penaranda, Hacène Khiri, Sabine Camiade, Lucie Molet, Melissa Lebsir, Anne Plauzolles, Laurent Chiche, Bernard Blanc, Edwin Quarello, Philippe Halfon

**Affiliations:** 1 Clinical Research and R&D Department, Laboratoire Européen, Alphabio - Biogroup, Marseille, France; 2 FCRIN INSERM US015, CHU de Toulouse, Hôpital PURPAN, Toulouse, France; 3 Bacteriology Department, Laboratoire Européen Alphabio - Biogroup, Marseille, France; 4 Infectious and Internal Medicine Department, Hôpital Européen Marseille, Marseille, France; 5 Gynecology Department, Hôpital Européen Marseille, Marseille, France; 6 Prenatal Screening and Diagnostic Unit, Obstetrics and Gynecology Department, St Joseph Hospital, Marseille, France; 7 Image2 Center, Marseille, France; Massachusetts General Hospital, UNITED STATES

## Abstract

**Background & aim:**

Screening for genital infection (GI) such as bacterial vaginosis (BV) and yeast infection, for sexually transmitted infection (STI), and for asymptomatic carriage of group B streptococcus (GBS) in pregnant women are common reason for medical appointments. The diagnosis and control of GIs, STIs, and GBS are major issues, for fertility and overall well-being of affected women. Conventional testing is performed using vaginal/cervical classical sampling (VCS); this procedure requires pelvic examination performed by health care professionals which raises concerns among women. Vaginal-self-sampling (VSS), as an alternative to VCS, might capture more women.

The aim was first to show non-inferiority of VSS compared with VCS to screen for GIs, STIs, and GBS; second to determine the feasibility of VSS.

**Methods:**

VSS and VCS from 1027 women were collected by health care professionals and simultaneously carried out on each patient. GIs, STIs, and GBS were systematically screened in both paired VSS and VCS samples. Non-inferiority of VSS compared with VCS was assessed using z statistic for binomial proportions.

**Results:**

Prevalence of GIs were 39.7% using VSS and 38.1% using VCS (p = 0.0016). Prevalence of STIs was 8.5% (VSS) vs 8.1% (VCS) (p = 0.0087). Prevalence of GBS was 13.4% (VSS) and 11.5% (VCS) (p = 0.0001). Most participants (84%) recommended the use of VSS.

**Conclusions:**

This study shows that VSS was not inferior to VCS for the detection of GIs, STIs, and GBS. This study provides evidence that VSS can be used as a universal specimen for detection of lower genital tract infections in women.

**Study identification number:**

ID-RCB 2014-A01250-4.

## Introduction

Common reasons for medical appointments are screening for genital infections (GI) such as bacterial vaginosis (BV) and yeast infection, screening for sexually transmitted infection (STI), and screening for asymptomatic carriage of group B streptococcus (GBS) in pregnant women. BV and yeast infection are the most common lower genital tract disorders among women of reproductive age [1, 2]. STIs are a major public health concern, with *Chlamydia trachomatis* (CT), *Neisseria gonorrhoeae* (NG), *Trichomonas vaginalis* (TV) and genital herpes (herpes simplex virus, HSV) the most prevalent STI and *mycoplasma genitalium* (MG) an emerging sexually transmitted pathogen [3, 4]. The diagnosis and control of GIs, BVs, and STIs are important issues, given their long-term consequences for fertility and overall well-being of affected women [1, 4]. In the same way, intrapartum screening for GBS colonisation is recommended in France at 35 to 37 weeks of gestation to initiate antibiotic prophylaxis and prevent early-onset infection in neonates [5, 6].

In current medical practice, vaginal and/or cervical sampling is indicated in cases of suspicion or screening for BV and yeast infection, STIs or cases of asymptomatic carriage in pregnant women in the eighth month of pregnancy. Conventional testing is performed by using vaginal and/or cervical classic sampling (VCS). This procedure requires a pelvic examination, speculum use and vaginal or endocervix sampling by health care professionals such as gynaecologists, general practitioners, medical biologists, and midwives. Concerns related to cultural and religious norms, as well as economic issues, are often cited as barriers to screenings performed with this method [7–10]. Moreover, specimen collection is often complicated by in-clinic difficulties, such as delayed waiting time to obtain a medical appointment and the lack of practitioners [11].

Vaginal self-sampling (VSS) has progressively emerged as an alternative to VCS, primarily for human papillomavirus (HPV)–associated cervical cancer screening. Previous studies have already been conducted by several teams, including us, and shown the efficacy and acceptability of VSS in this indication [12–17]. More recently, VSS was recommended by attendees of a National Institutes of Health (NIH) workshop for CT and NG screening [8, 18]. Indeed, the ease and acceptability of vaginal self-sampling could facilitate follow-ups and potentially help in the prevention of gynaecological disorders.

By performing a large, cross-sectional study, our objectives were first to show non-inferiority of VSS compared with VCS for the detection of GI such as BV and yeast infection STI infections and GBS asymptomatic carriage in pregnant women; and second to determine the feasibility of self-collection by women requiring vaginal and/or cervical sampling for their gynaecological monitoring.

## Patients and methods

### Sample size calculations

Three sample sizes were calculated to demonstrate non-inferiority of VSS compared with VCS for the detection of each infection separately (GIs, STIs, and GBS). STI was the infection type that requires the most patients to demonstrate noninferiority of VSS compared with VCS; considering a prevalence of 4.4% [95% Confidence Interval (95% CI) 2.1–8.0%] for VCS (P_VCS_) (results observed in routine laboratory analysis from 225 patients in 2014), and a non-inferiority margin (M) of 2.3% (defined as the difference between P_VCS_ and 95% CI lower bound of P_VCS_). Under these conditions, if there is no difference between P_VCS_ and P_VSS_, then at least 984 patients were required to be sure that the upper limit of a one-sided 95% CI excluded a difference in favour of VCS of more than 2.3% (alpha level of 5%, and statistical power of 80%) [19]. Using a similar approach, 981 patients were required for GIs, and 708 for GBS. Overall, at least 984 were needed to demonstrate noninferiority of VSS compared with VCS.

### Study population

From October 2015 to March 2018, 1067 women were proposed to participate in this study; 39 refused (18 did not formulate any reason to decline participation, eight did not think they could do the self-swabbing correctly, four did not understand French language, and the remaining nine patients did not have enough time to participate. Overall, 1028 participants, including 224 pregnant women (21.8%), were recruited from 11 clinical centres in Marseille, France. Recruitment was conducted by 26 clinical practitioners including gynaecologists and midwives.

Women were eligible to participate if they were 18–65 years of age and if they presented vaginal/cervical sampling indications: suspicion or screening for GI (BV or yeast infection) and/or STI and/or asymptomatic carriage of GBS). At recruitment, clinical practitioners filled out a clinical information form where sampling indications were mentioned. In addition, participants were asked to complete a questionnaire to collect their feedback toward self-sampling and classic sampling (S1 Questionnaire)(one patient did not answered the questionnaire).

Written informed consents were obtained from the participants at recruitment. The study was sponsored by the Hôpital Européen Marseille Clinical Research Department in collaboration with the Clinical Research and R&D Department of the Laboratoire Européen Alphabio. This biomedical clinical study was authorised by the French competent authority (ANSM, www.ansm.sante.fr) and received the agreement of an ethics committee (CPP Sud Méditerranée I). The French national identification number of the study is ID-RCB 2014-A01250-47.

### Sample collection

**Fig 1:**
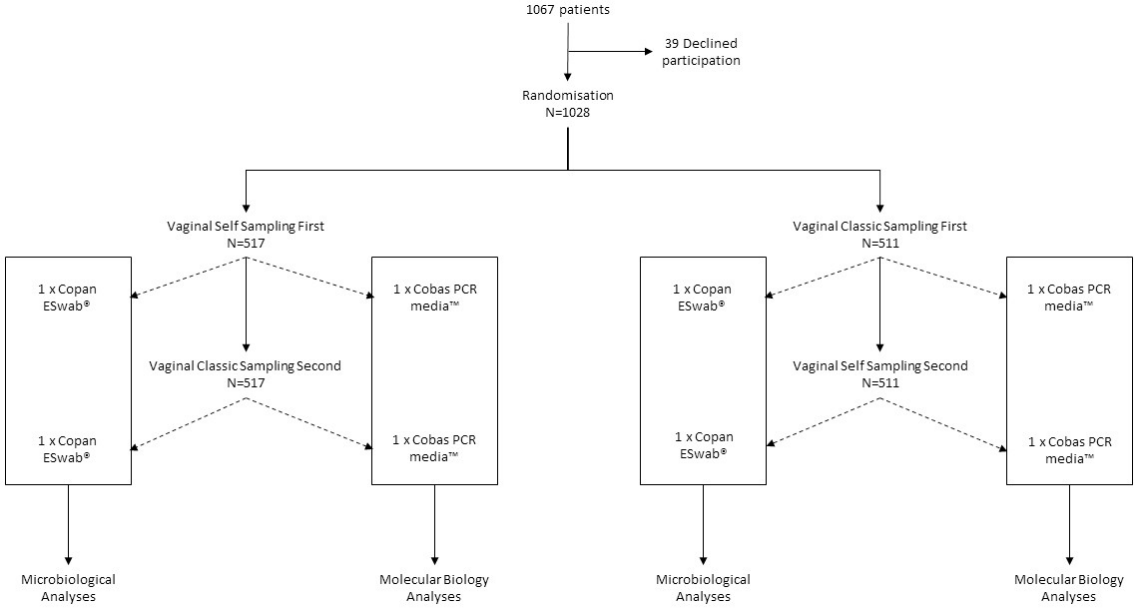
Study flowchart.

Randomised sampling technique was performed as follows: half of the women were asked to perform VSS before VCS, and the other half were asked to perform VSS after VCS (Fig 1). Sampling order was specified on the clinical information form. A VSS paired sampling (randomised order) and a VSS acceptability survey were prospectively collected.

Written and schematic instructions were provided to patients before VSS. Performing their VSS, participants were instructed to push the swab gently into their vagina, turn it two times while wiping the vaginal wall and slowly remove the swab. The swab was then inserted and released into the collection tube prior to capping with the lid. VCSs were collected following the same procedure under speculum examination. VSS and VCS were collected at the same time at the clinic.

Two swabs were collected for both VSS and VCS (four overall for each patients): two using Copan ESwab^®^ (Biomérieux, Marcy l’Etoile, France) and two using Cobas PCR media^™^ (Roche Diagnostics, Meylan, France). Microbiological analysis and HSV molecular biology analysis were performed using Copan ESwab, and TV, CT, NG and MG molecular biology analysis were performed using Cobas PCR media.

### Microbiological analysis

The VSS and VCS tubes were shipped at ambient temperature to Alphabio laboratories in Marseille for microbiological analysis and STI testing. Both VSS and VCS were processed within 24 h of sample collection. For each of the paired samples, routine microbiology analysis was performed: BV was defined by a Nugent score equal or superior to 7, and yeast infection was defined by yeast growth on nutrient agar media [20]. Gram stain and yeast culture were performed on the two Copan ESwabs (one swab for each sampling randomization sequence). Each swab were smeared on a slide and sent for culture.

In addition, DNA was extracted from a 3 ml Cobas PCR Media^™^ sampling tube using Cobas 4800 (Roche Diagnostics): 700 μl are needed for CT, NG, and HSV analyses. Remaining eluted sample obtained from Cobas 4800 (approximately 80 μl) was then used for MG and TV analyses. CT and NG analyses were performed using Cobas^®^ (Roche Diagnostics); MG and TV were performed using TIB MOLBIOL LightMix^®^ kit (Roche Diagnostics), and HSV types 1and/or 2 using Cobas 4800^®^ (Roche Diagnostics).

### Statistical analysis

Data were reported using frequencies and proportions. Non-inferiority of VSS compared with VCS was assessed using z statistic for binomial proportions. The null hypothesis for the non-inferiority test is H0: P_VSS_−P_VCS_ ≤-M versus the alternative H1: P_VSS_−P_VCS_ >-M, where P_VSS_ stands for the prevalence observed for VSS, P_VCS_ stands for the prevalence observed for VCS and M stands for the non-inferiority margin defined as the 95% CI lower bound of P_VCS_. Exact Clopper–Pearson CI95s were reported. Overall agreement between VCS and VSS was reported, calculating the rate of concordant cases, kappa statistic, positive likelihood ratio (LR+), negative likelihood ratio (LR-), sensitivity (Se) and specificity (Sp) (all with their 95% CI) [21]; the McNemar test for matched paired data was computed to test the marginal homogeneity between VCS and VSS; McNemar test is used for within-subject designs where the change of a dichotomous measure (here positivity/negativity) is assessed across two within-subjects observations; the proportions of discordant cases (subjects positive with VCS and negative with VSS, or negative with VCS and positive with VSS) dictates statistical significance. Sampling randomisation sequences were compared using the Breslow-Day test (VCS before VSS vs. VSS before VCS) assuming equality of odds ratios (OR) (null hypothesis). Statistical tests were assessed using a significance criterion of α = 0.05. Statistical computations were performed using SAS v. 9.4 software (SAS Institute, Inc., Cary, NC).

## Results

### Sampling indications

Mean age was 35.2 (standard deviation 11.5) years old. Indications of genital sampling comprised suspicion of GI in 380 participants (BV or yeast infection), suspicion of STI in 62 participants, suspicion of GI and STI in 419 participants, and GBS asymptomatic carriage in 166 pregnant women (Table 1).

**Table 1:** Characteristics of surveyed women.

Characteristics	Value
Age—Mean (Sd) (years)	35.2 (11.5)
Pregnancy—N (%)	224 (22%)
Sampling indication—N (%)	
*Suspicion of GI*	380 (37%)
*Suspicion of STI*	62 (6%)
*Suspicion of GI + suspicion of STI*	420 (41%)
*Screening of asymptomatic carriage of group B streptococci*	166 (16%)
Type of preferred sampling method—N (%)*	
*Self-sampling*	322 (31%)
*Classical sampling*	268 (26%)
*Both*	437 (43%)
Reasons to prefer self-sampling—N (%)	
*Easy to use*	309/322 (96%)
*In accordance with religious and cultural norms*	69/322 (21%)
*Promote the monitoring of GI*	128/322 (40%)
*Cheaper alternative*	20/322 (6%)
*Other reasons*	22/322 (7%)
Reasons to prefer classical-sampling—N (%)	
*Medical assistance*	244/268 (91%)
*Embarrassment*	64/268 (24%)
*Hard to process self-sampling*	35/268 (13%)
*Physical discomfort*	29/268 (11%)
Considering that medical appointment hinders gynaecologic monitoring—N (%)*	
*Yes*	278 (27%)
*No*	749 (73%)
Considering that VSS enhances gynaecologic monitoring—N (%)*	
*Yes*	579 (56%)
*No*	448 (44%)
Recommend the use of self-sampling—N (%)*	
*Yes*	867 (84%)
*No*	160 (16%)

*one patient did not answered the questionnaire.

### Detected infections using vaginal self-sampling and concordance with vaginal classical sampling

Concordance between VSS and VCS was assessed among 1026 samples as one VCS failed for molecular biology analysis. Prevalence of overall GIs was 39.7% using VSS and 38.1% using VCS (non-inferiority p = 0.0016), prevalence of STIs was 8.5% using VSS and 8.1% using VCS (non-inferiority p = 0.0087), and prevalence of GBS was 13.4% using VSS and 11.5% using VCS (non-inferiority p = 0.0001, showing no inferiority of VSS to VCS (Table 2).

**Table 2:** Detection rates of GI, STI infection and GBS asymptomatic carriage, and non-inferiority test.

Sample type	Vaginal classic-sampling		Vaginal self-sampling		P-value (for non-inferiority of vaginal self-sampling)
	Positive N	% (95% CI)	Positive N	% (95% CI)	
**GI**	392	38.1 (35.2–41.1)	408	39.7 (36.7–42.7)	0.0016
*Bacterial vaginosis*	*105*	*10*.*7 (8*.*8–12*.*7)*	*104*	*10*.*6 (8*.*7–12*.*5)*	*0*.*0348*
*Yeast infection*	*304*	*29*.*6 (26*.*8–32*.*4)*	*322*	*31*.*3 (28*.*5–34*.*2)*	*0*.*0009*
**STI**	83	8.1 (6.4–9.7)	87	8.5 (6.8–10.2)	0.0087
*Trichomonas vaginalis*	*18*	*1*.*8 (1*.*0–2*.*6)*	*19*	*1*.*9 (1*.*0–2*.*7)*	*0*.*0217*
*Chlamydia trachomatis*	*33*	*3*.*2 (2*.*1–4*.*3)*	*34*	*3*.*3 (2*.*2–4*.*4)*	*0*.*0354*
*Neisseria gonorrhoeae*	*9*	*0*.*9 (0*.*3–1*.*4)*	*10*	*1*.*0 (0*.*4–1*.*6)*	*0*.*0140*
*Mycoplasma genitalium*	*14*	*1*.*4 (0*.*7–2*.*1)*	*14*	*1*.*4 (0*.*7–2*.*1)*	*0*.*0336*
*Herpes simplex virus*	*17*	*1*.*7 (0*.*9–2*.*4)*	*19*	*1*.*9 (1*.*0–2*.*7)*	*0*.*0120*
**Group B streptococcus**	118	11.5 (9.5–13.2)	138	13.4 (11.3–15.5)	0.0001
*Group B streptococcus in pregnant women*	*18*	*8*.*0 (4*.*5–11*.*6)*	*20*	*8*.*9 (5*.*2–12*.*7)*	*0*.*0010*

Primary outcomes are in bold; complemental outcomes are in italic; Vaginosis detection rates were determined among patients with bacterial vaginosis and/or yeast infection. STI detection rates were determined among patients with TV, and/or CT, and/or NG, and/or MG, and/or HSV infection.

Prevalence of BV was 10.6% using VSS and 10.7% using VCS (p = 0.0348), prevalence of yeast infection was 31.3% using VSS and 29.6% using VCS (p = 0.0009). Prevalence of specific STIs were 1.9% using VSS and 1.8% using VCS for TV (p = 0.0217), 3.3% using VSS and 3.2% using VCS for CT (p = 0.0354), 1.0% using VSS and 0.9% using VCS for NG (p = 0.0140), 1.4% using VSS and 1.4% using VCS for MG (p = 0.0336), and 1.9% using VSS and 1.7% using VCS for HSV (p = 0.0120); and kappa values computed for STI were all superior or equal to 0.95, which indicates excellent agreement between self-collected and physician-collected samples (Table 3).

**Table 3:** Agreement between VSS and VCS for the detection of GI agents.

Sample	Result	VSS		Overall Agreement–% (95% CI)	Kappa	LR+**	LR-**	Se**	Sp**	P-value (McNemar)
	VCS				(95% CI)	(95% CI)	(95% CI)	% (95% CI)	% (95% CI)	
GI	+	350	42	90.3%	0.80	12.66	0.15	89.3%	90.9%	0.1096
	-	58	578	(88.3–92.0)	(0.76–0.83)	(9.43–17.00)	(0.12–0.19)	(86.2–92.4)	(88.7–93.1)	
Bacterial vaginosis	+	83	22	95.7%	0,77	31.90	0.20	79.0%	97.7%	0.7576
	-	20	849	(94.2–96.9)	(0,71–0,84)	(20.89–48.72)	(0.13–0.30)	(71.2–86.8)	(96.7–98.7)	
Yeast infection	+	273	31	92.2%	0.82	19.31	0.16	89.8%	93.2%	0.0442
	-	49	675	(90.4–93.8)	(0.78–0.85)	(13.64–27.33)	(0.12–0.21)	(86.4–93.2)	(91.4–95.0)	
Overall STI	+	82	1	99.4%	0.96	886.92	0.06	98.8%	99.5%	0.1025
	-	5	940	(98.7–99.8)	(0.93–0.99)	(124.97–6294.30)	(0.02–0.13)	(96.5–100)	(99.1–99.9)	
TV	+	9	0	99.8%	0.97	NA	0.18	100%	99.8%	0.3173
	-	2	1017	(99.3–100)	(0.92–1.00)		(0.05–0.64)	(NA)	(99.5–100)	
CT	+	33	0	99.9%	0.98	NA	0.03	100%	99.9%	0.3173
	-	1	994	(99.5–100)	(0.95–1.00)		(0.00–0.20)	(NA)	(99.7–100)	
NG	+	6	1	99.9%	0.95	1022	0.00	85.7%	100%	0.3173
	-	0	1021	(99.5–100)	(0.84–1.00)	(144.10–7248.56)		(59.8–100)	(NA)	
MG	+	14	0	100%	1.00	NA	0.00	100%	100%	NA
	-	0	1014	(N/A)				(NA)	(NA)	
HSV	+	16	1	99.6%	0.89	848.00	0.16	94.1%	99.7%	0.3173
	-	3	1006	(99.0–99.9)	(0,78–1,00)*	(118.42–6072.70)	(0.06–0.45)	(82.9–100)	(99.4–100)	
GBS	+	112	6	96.7%	0.86	120.39	0.19	94.9%	97.1%	0.0004
	-	26	884	(95.6–97.9)	(0.81–0.91)	(54.01–268.33)	(0.13–0.27)	(90.9–98.9)	(96.0–98.2)	
GBS in pregnant women	+	17	1	98.2%	0.89	173.40	0.15	94.4%	98.5%	0.3173
	-	3	203	(95.5–99.5%)	(0.77–1.00)	(24.33–1235.76	(0.05–0.43)	(72.7–99.9)	(95.8–99.7)	

**LR+, LR-, sensitivity and specificity of VSS according to VCS.

*Agreement between VSS and VCS were determined among 1026 patients (two VCS failed and was excluded from statistical analysis).

Prevalence of GBS asymptomatic carriage was 8.9% using VSS and 8.0% using VCS in pregnant women (p = 0.0010).

Agreements between VCS and VSS remained high ranging from 90.3% for GI up to 98.3% for GBS asymptomatic carriage in pregnant women, and kappa statistics showed substantial agreement between VCS and VSS (ranging from 0.77 for BV to 0.89 for GBS asymptomatic carriage in pregnant women). McNemar test for marginal homogeneity was not significant except for yeast infection and GBS (p = 0.0442 and p = 0.0004, respectively).

No significant difference was observed between ORs of sampling randomisation sequences among GI agents; assuming that the probability of detecting a specific GI agent was not different whatever randomisation sequence (VCS before VSS vs. VSS before VCS) (Table 4).

**Table 4:** Comparison of sampling randomisation sequences among GI agents.

	VCS before VSS	VSS before VCS	P (Breslow-Day)
	OR (95% CI)*	OR (95% CI)*	
GI	78 (44–141)	89 (49–162)	0.7652
Bacterial vaginosis	95 (41–222)	356 (118–1068)	0.0605
Yeast infection	136 (69–268)	109 (57–209)	0.6459
Overall STI	5724 (581–56439)	10649 (947- >100000)	0.2580
HCV	2024 (166–24657)	2000 (164–24365)	0.9947
GBS	855 (191–3832)	542 (167–1763)	0.6360
GBS in pregnant women	1100 (64–18844)	309 (24–3909)	0.6000

*Odds ratio with 95% confidence interval.

### Attitude toward self-sampling and clinical collection

322 (31%) women preferred VSS and 268 (26%) preferred VCS (p = 0.045) (43% did not have a preference (Table 1). The primary reason women gave for preferring VSS was its ease of use: 96% (309/322). Other reasons to prefer VSS were as follows: 40% (138/322) of them estimated that VSS facilitated monitoring of GI, 21% considered VSS in accordance with their cultural norms and 6% considered VSS a cheaper alternative to VCS. The main reason women preferred VCS was because they preferred to be sampled by a practitioner: 91% (244/268).

Of the 1027 surveyed participants, 84% (867) would recommend the use of VSS, and 56% (579) asserted that VSS instead of VCS would encourage them to be monitored more regularly. In contrast, only 27% (278) of participants considered the need to have a medical appointment to perform vaginal sampling as an obstacle to gynaecological monitoring.

## Discussion

This study is the first large-scale cross-sectional study conducted to evaluate the efficacy of VSS, with more than 1000 participants in a global clinical setting, which also included pregnant women. Efficacy of VSS was evaluated for a large panel of infectious agents. VSS was comparable with VCS for CT, NG, MG and TV detection by NAAT, consistent with findings in other cohorts [10, 18, 22–26]. CT, NG, MG and TV detection rates were lower than those previously reported in under-screened low-income women [4, 27] and might be influenced by recruitment. In fact, women participating in this study were recruited by clinicians during their course of care and STIs were suspected in only 6% of participants, as has been mentioned before. HSV detection rates were also very low, consistent with intermittent viral shedding in the mucosa of infected populations [28]. The close agreement (99.6%; [99–99.9]) between VSS and VCS detection rates encourages the use of VSS to detect any HSV active infection.

GI and GBS asymptomatic carriage were screened by microbiological analysis. The microbiological analysis implies another issue compared to NAAT: it should be rapidly performed to maintain bacterial viability and avoid false negative results. In this study, both VSS and VCS were processed within a maximum of 24 hours, thus ensuring good analytical conditions. Prevalence of bacterial vaginosis and yeast infection were similar to previously reported [29–37], and self-collection was comparable to clinician-based collection. These results showed that self-collected vaginal swabs are an effective method for the diagnosis of both bacterial vaginosis and yeast infection. Some papers support the existing knowledge regarding GBS screening by VSS; several authors showed the non-inferiority of VSS compared to VCS [1, 38–41]. One study reported contradictory results, demonstrating that self-collection was less sensitive to detect GBS carriage [42]. In this study good kappa values were reported, eg. 0.86 and 0.89 in pregnant women, establishing the good reliability of self-collected vaginal swabs to screen GBS asymptomatic carriage. In addition, sensitivity was 94.4% in pregnant women, allowing the use of VSS in this indication. A difference was observed between VSS and VCS for yeast infection and GBS screening. In both cases, VSS was more sensitive than VCS. For GBS screening one explanation might be the sampling method of the perineum: to optimize sensitivity US guidance recommends universal screening (using a vaginal and a rectal swab) for GBS colonization [43]. For yeast infection, due to both digestive and vaginal colonization, discordant findings might not be directly linked to the type of sampling.

Given its excellent diagnostic performance, especially for high NPV, health care practitioners can be confident in the use of VSS to detect any symptomatic or asymptomatic infection.

The principal indication was suspicion of GI (bacterial and yeast infection), which represented 77.8% (800) of the study’s participants. GI screening was combined with STI screening in 420 women (52%). In fact, GI, particularly BV, have been found to be associated with sexually transmitted agents such as NG, CT and MV [1, 44]. The second indication was GBS screening in pregnant women. It represented 166 (16.2%) of analysed samples, which represented 74% of pregnant women included in the study. Finally, STI screening was requested only in 6% of participants.

Because recruitment was conducted directly by health care practitioners, it was not surprising to find good adhesion to clinician examination among the surveyed women. In fact, only a minority (27%) considered the consultation an obstacle to seeking care. However self-collection was well accepted and 84% of participants would recommend the use of VSS. Moreover, 56% of surveyed women stated that VCS substitution by VSS would ensure better follow-up. Only 26% of participants preferred VCS to VSS, mostly because they were not confident with the self-collection process. A recent study showed that VSS acceptability improves with age and the need for attraction strategies that are more appealing to younger women [45]. We can therefore assume that the proportion of women who prefer VSS will increase over time. As previously reported, acceptability of VSS was satisfactory among sexually active women surveyed in this study [46–50].

Conversely to most studies including small subsets of participants, our study benefited from a large cohort of women, hence providing an accurate assessment of the added value of VSS for future clinical standard-of-care. This large cohort provided robust assessment of diagnostic performance. In addition, all self-collection results were paired with vaginal classical co-testing. Both samples were collected with the same procedure, treated with the same analytical methodology and within the same delay.

Our recruitment approach excluded infrequently screened women, although previous studies have already reported a similar VSS efficacy and acceptability within that population [8, 18]. The novelty of our study focuses on the utility of VSS to improve gynaecologic monitoring regarding the challenge of pelvic examination.

The study could have been strengthened by assessing VSS adequacy (ie β-globin testing), although high agreements between VCS and VSS comforted its validity.

Another limitation of our study is that α-risk was not corrected for multiplicity. However, this limitation should be relativized according to the design of the study (non-inferiority of VSS vs. VCS), the fact that observed absolute prevalence’s of VSS are higher than VCS il all cases (except for BV), and that all p-value are less than 0.05 (among which only few are between 0.01 and 0.05)[51, 52]

Since 2014, in the United States, the NIH and the Centres for Disease Control and Prevention (CDC) recommend the use of VSS for screening of CT and NG by nucleic acid amplification techniques [8, 53]. In addition, they encourage the research community to initiate clinical trials and obtain evidence needed to use VSS for GI screening in a wide variety of settings. Given its efficacity and acceptability, VSS may be considered as an effective alternative to alleviate gynaecological consultation among women needing vaginal sampling for clinical reasons, antenatal screening, and sexual health examinations. Moreover, VSS may enhance acceptability among under-screened low-income women and thereby improve detection and treatment of GIs and STIs. Another interesting application of VSS would be the reliability to repeat a positive test to ensure clearance of the infection following treatment.

This study shows that VSS was not inferior to VCS for the detection of GIs, STIs, and GBS. It remains the most exhaustive in screening bacterial and yeast infections, multiple STI agents and asymptomatic GBS carriage. Throughout constant efforts to improve medical care, VSS seems to be a viable alternative to the classic physician sampling. It may be a good option to enhance gynaecological monitoring while also alleviating the need for patient intimacy. This study provides evidence that VSS can be used as a universal specimen for detection of lower genital tract infections in women [8].

## Supporting information

S1 Questionnaire (DOCX)

S1 Data (CSV)
