# Supplementary material for: Acceptability and efficacy of vaginal self-sampling for genital infection and bacterial vaginosis: A cross-sectional study
Source: PLoS One. 2021 Nov 18;16(11):e0260021. doi: 10.1371/journal.pone.0260021 (PMC8601421; doi:10.1371/journal.pone.0260021)
Supplement: S1 Questionnaire — (DOCX) [file pone.0260021.s001.docx]

**Supplementary File 1**

**APV Protocol**

**Questionnaire about vaginal self-sampling**

**1-Which type of sampling did you preferred?**

□ Vaginal Self-Sampling, why? (thick one or more boxes)

□ Easy and less embarrassing

□ Is not opposed to my cultural norms

□ Facilitate monitoring of genital infections

□ Financial reason (free of charge)

□ Other:

□ Vaginal Classic-Sampling, why? (thick one or more boxes)

□ The practitioner has more experience than I do, and the sampling may be of better quality

□ Feel not comfortable with vaginal self-sampling

□ Vaginal self-sampling was more unpleasant or painful than classic sampling

□ It was hard to perform vaginal self-sampling

□ Both

**2-Accordinf to you, is a medical appointment to perform vaginal sampling is an obstacle to gynecological monitoring? (thick only one box)**

□ Yes; do you prefer that your vaginal sampling is performed by: (thick one or more boxes)

□ Medical biologist at the medical laboratory?

□ Yourself at the medical laboratory?

□ Yourself at home?

□ No

**3-Would you recommend vaginal self-sampling?**

□ Yes

□ No

**4-In the case where vaginal self-sampling replaces classical sampling, would it encourage you to be monitored more regularly?**

□ Yes

□ No

**5-What is your highest level of education?**

□ None (elementary school)

□ Basic (secondary school level)

□ Medium (high school level)

□ High (university level)
